# Supplementary material for: Copper bioavailability is a KRAS-specific vulnerability in colorectal cancer
Source: Nat Commun. 2020 Jul 24;11:3701. doi: 10.1038/s41467-020-17549-y (PMC7381612; doi:10.1038/s41467-020-17549-y)
Supplement: Supplementary file 1 — Supplementary Information [file 41467_2020_17549_MOESM1_ESM.pdf]

Supplementary information

Copper bioavailability is a KRAS-specific vulnerability in colorectal cancer

Aubert *et al.*

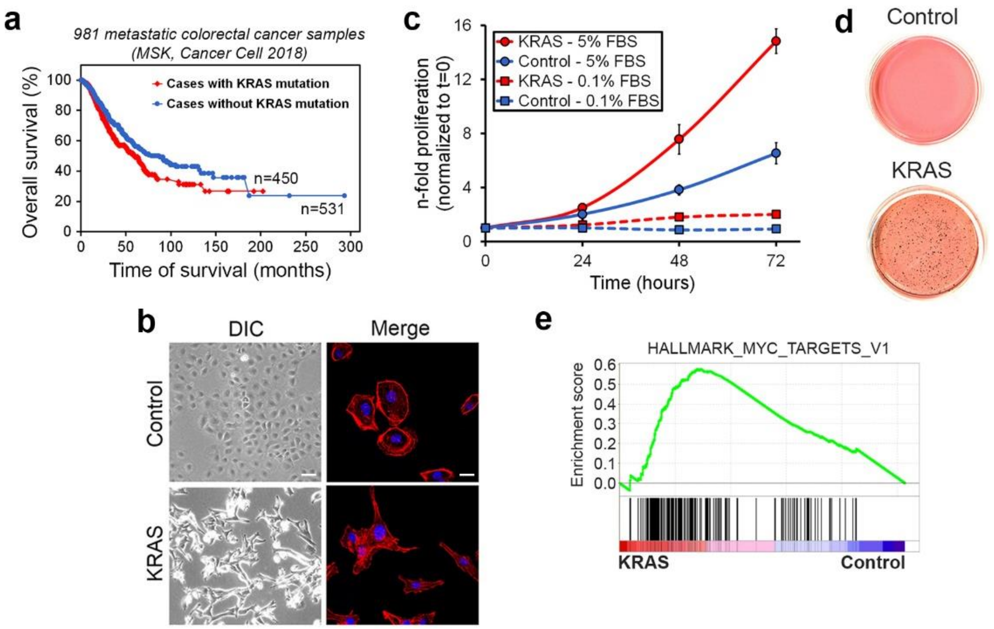

Supplementary Figure 1: Validation of intestinal epithelial cell model for KRAS-mediated transformation. a, Kaplan-Meier plot comparing overall survival of metastatic colorectal cancer patients with or without *KRAS* mutations from publicly available data analyzed on the cBioPortal database for Cancer Genomics (https://www.cbioportal.org/study/summary?id=crc_msk_2017). **b-d,** Control and KRAS IEC-6 cells were analyzed for the following: **b,** cellular phenotype with phase-contrast microscopy (DIC) [scale=10 µm], Phalloidin (red) and DAPI (blue) [scale=20 µm]; **c,** cellular proliferation using WST-1 assays in medium containing 0.1% or 5% FBS. Data were normalized to initial seeding (t=0) and expressed as mean ±SD of four replicates per condition; and **d,** adherence-independent growth by soft agar assays. Data represent n=3 independent experiments. **e,** Analysis of Control and KRAS IEC-6 transcriptome dataset with GSEA database correlated with upregulation of hallmark gene set "MYC_TARGETS_V1". Source data are provided as a Source Data file.

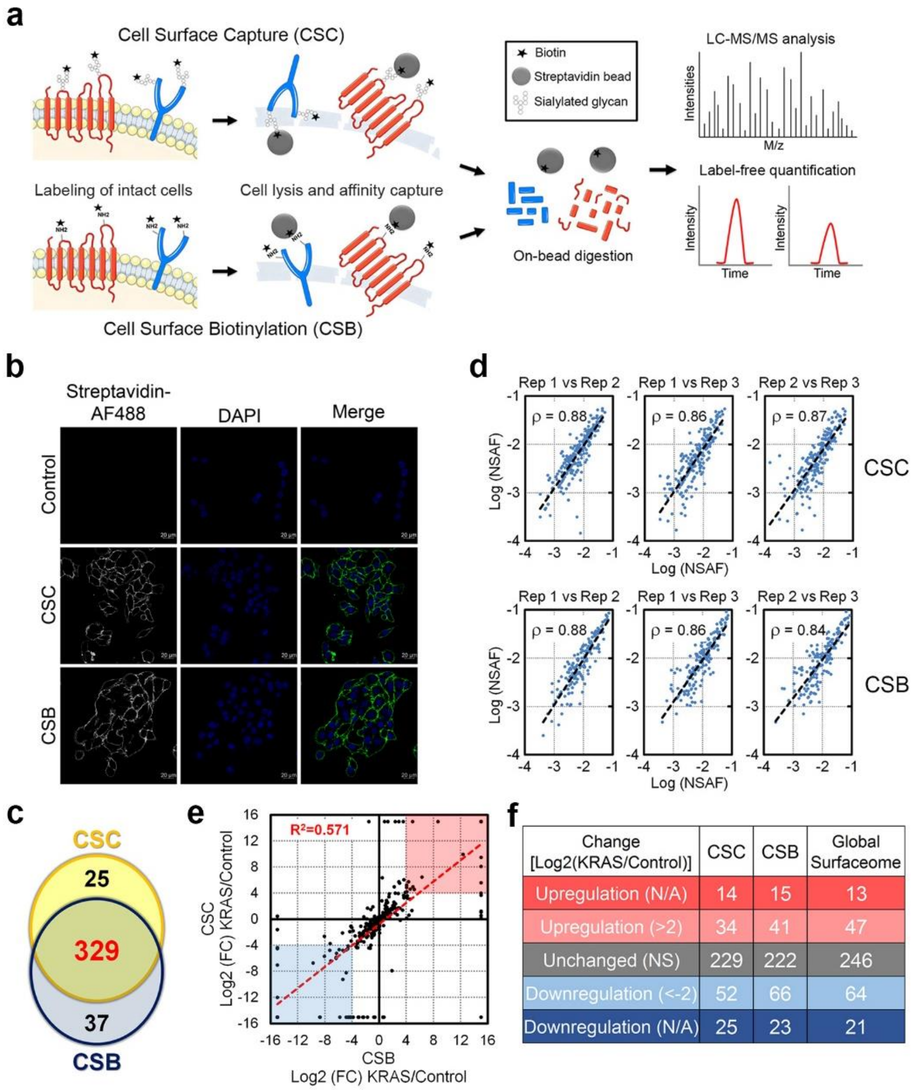

Supplementary Figure 2: Validation of the surface proteomics protocol to quantify cell-surface proteins. a, Schematic outline of the surfaceomic approach optimized for the identification and quantification of cell-surface proteins. Cell Surface Capture (CSC) and Cell Surface Biotinylation (CSB) methods. **b,** Immunofluorescence (IF) images showing the specificity of cell-surface labeling with both CSC and CSB techniques, scale=20 μm. Data represent n=3 independent experiments. **c,** Venn diagram of all high-confidence cell-surface proteins identified in the KRAS-regulated surfaceome of IEC-6 cells, isolated using CSC or CSB. **d,** Comparison of Normalized Spectral abundance Factor (NSAF) to determine the reproducibility in protein

identifications between triplicates for each of the biotinylation method. **e,** Graph deciphering the correlation between CSC and CSB for the quantification of differentially expressed cell-surface proteins by KRAS (R^2=0.571). Data are expressed as Log_2 FC (KRAS/Control). **f,** Table summarizing the number of proteins significantly upregulated (red) (Log_2 FC $\geq$ 2) or downregulated (blue) (Log_2 FC $\leq$ -2) at the cell-surface of IEC-6 KRAS compared to Control cells. N/A (non applicable) stands for proteins identified uniquely in Control or KRAS cells. Source data are provided as a Source Data file.

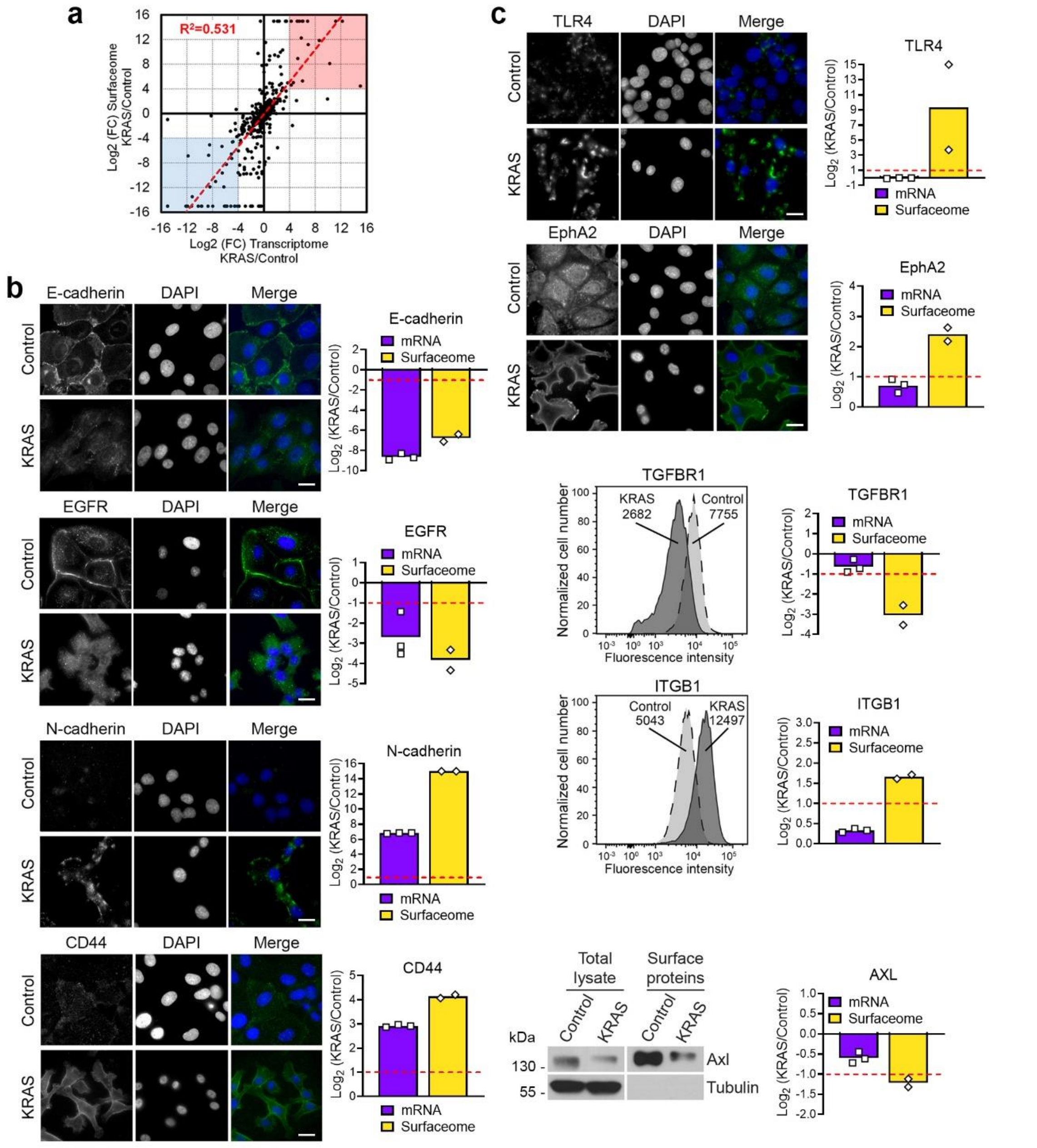

Supplementary Figure 3: Validation of cell-surface proteins differentially modulated by mutant KRAS. a, Scatter plot showing the correlation between surfaceome and transcriptome datasets (R^2=0.531). Data are expressed as Log_2 FC (KRAS/Control). **b-c,** IF, flow cytometry, or immunoblot (IB) images showing the cellular localization of transcription-dependent upregulated (CDH2, CD44) or downregulated (CDH1, EGFR) (**b**), and transcription-independent upregulated

(TLR4, EPHA2, ITGB1) or downregulated (TGFβR1, AXL) cell-surface proteins (**c**), in KRAS *versus* Control cells. Graphs on the right illustrate the Log_2 FC (KRAS/Control) from the transcriptome (purple) and surfaceome (yellow) datasets. Red dashed lines indicate cut-off values statistical significance. Data are expressed as means of n=3 (mRNA) or n=2 (surfaceome) independent replicates per condition. All validation data represent n=3 independent experiments, scale=17 µm. Source data are provided as a Source Data file.

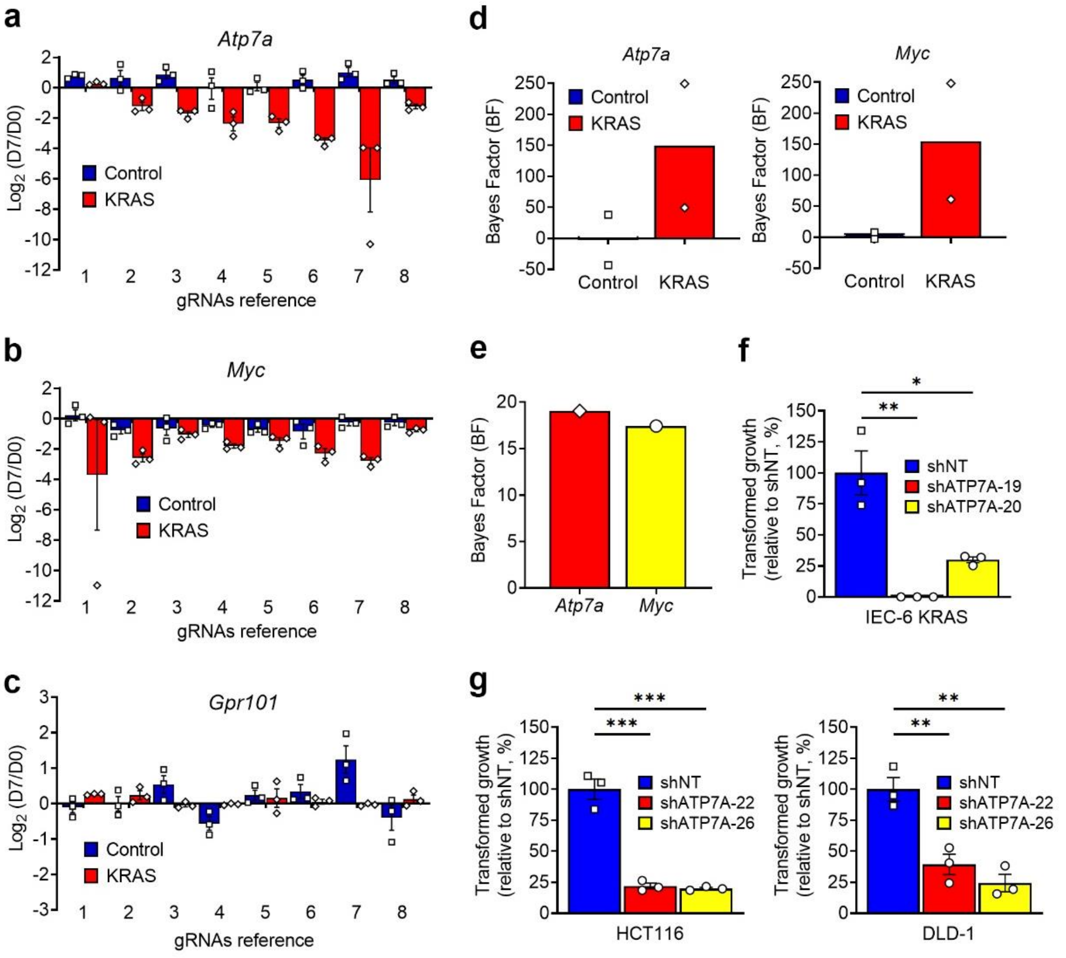

Supplementary Figure 4: ATP7A is essential for growth of KRAS-mutant cells. a-c, Graphs depicting the relative abundance of each of the eight gRNAs used in CRISPR/Cas9 screens in Control (blue) and KRAS (red) represented as Log_2 FC (Day 7/Day 0) for *Atp7a* (**a**), essential gene *Myc* (**b**) and non-essential gene *Gpr101* (**c**). Data represent n=3 replicates per condition and are representative of two independent CRISPR screens (**a-c**). **d,** Bar graphs depicting the Bayes Factor (BF) scores for *Atp7A* (left) and *Myc* (right), expressed as mean of the two *in vitro* CRISPR/Cas9 screens in Control and KRAS cells. **e,** Same as in **d,** but for *in vivo* CRISPR/Cas9 screen in KRAS IEC-6 xenografts. **f,** Adherence-independent growth on soft agar for KRAS IEC-6 cells with NT or ATP7A shRNAs (19 and 20), **P*=0.0174; ***P*=0.0049. **g,** Same as in **f,** except that experiments

were performed using HCT116 (left) and DLD-1 (right) with NT or ATP7A shRNAs (22 and 26), ***P=0.0008 (shNT *versus* shATP7A-22, HCT116); ***P=0.0006 (shNT *versus* shATP7A-26, HCT116); **P=0.0084 (shNT *versus* shATP7A-22, DLD-1); **P=0.0030 (shNT *versus* shATP7A-26, DLD-1). Data represent n=3 independent experiments per condition (**f, g**). Data and error bars represent mean ±SEM, and statistical significance is determined using unpaired two-tailed Student's t tests. *$P < 0.05$; **$P < 0.01$; ***$P < 0.001$. Source data are provided as a Source Data file.

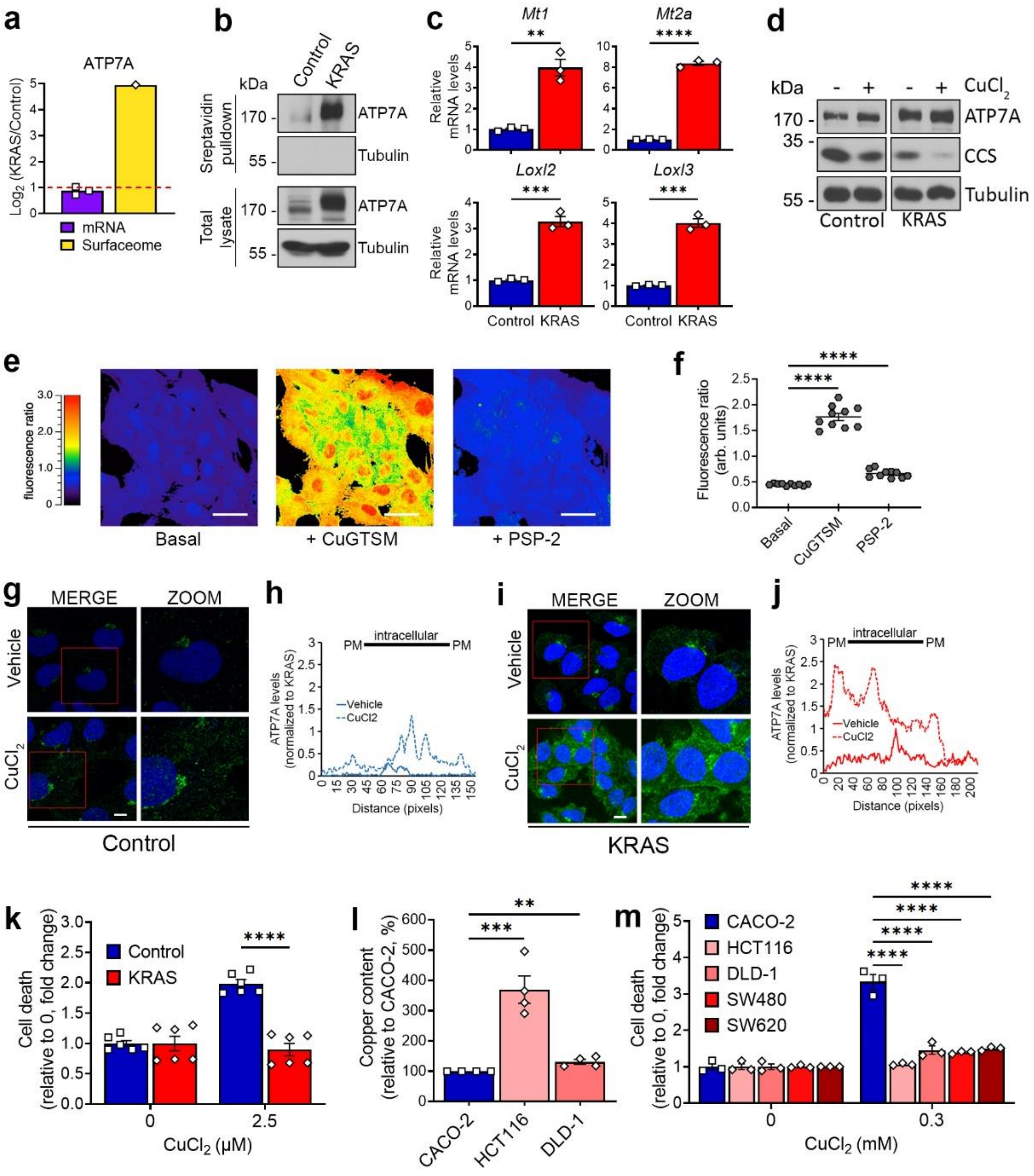

Supplementary Figure 5: ATP7A expression and Cu-export function are essential for survival of KRAS-mutant cells. a, Graph indicating the Log_2 FC (KRAS/Control) from the transcriptome (purple) and surfaceome (yellow) datasets for ATP7A. Red dashed line indicates cut-off value for statistical significance. Data are expressed as mean of n=3 (mRNA) or n=2 (surfaceome) independent replicates per condition. **b,** Biotinylated cell-surface proteins from Control and KRAS cells were processed for streptavidin pull-down. Eluted proteins and pre-

elution lysates were analyzed by IB for ATP7A and Tubulin. Data represent n=3 independent experiments. **c,** Relative mRNA expression levels of four Cu-dependent genes in Control and KRAS cells. Data represent n=3 independent experiments with two technical replicates. **d,** Control and KRAS cells were treated with or without $CuCl_2$ for 24h, and whole-cell lysates were analyzed by IB for ATP7A, CCS and Tubulin. Data represent n=3 independent experiments. **e,** Representative images of KRAS cells incubated with the fluorescence sensor crisp-17 and left untreated (Basal), treated with CuGTSM, or treated with high-affinity Cu chelator (PSP-2) in the presence of CuGTSM. Data represent n=3 independent experiments, scale=30 µm. **f,** Dot plot depicting the quantifications of crisp-17 fluorescence ratios averaged over n=10 cells. **g-j,** Same as **d,** except that ATP7A (green) was assessed by IF with DAPI (blue). Representative images for Control (**g**) and KRAS (**i**) cells, and the corresponding graphical distribution of ATP7A levels for Control (**h**) and KRAS (**j**). Data represent three random images from n=3 independent experiments, scale=10 µm. Data are normalized to untreated KRAS cells. **k,** Control and KRAS IEC-6 were treated with the indicated doses of $CuCl_2$ for 24h and processed for Annexin V staining by Flow cytometry. Data represent n=6 independent replicates. **l,** Measurement of total Cu levels for the indicated CRC cells as measured by ICP-MS analysis. Data represent n=4 independent experiments, ***P=0.0010 (CACO-2 *versus* HCT116); **P=0.0084 (CACO-2 *versus* DLD-1). **m,** Same as **k,** but for KRAS wild-type (CACO-2) and KRAS mutant (HCT116, DLD-1, SW480, SW620) CRC cells. Data represent n=3 independent experiments. For panels (**c, f, k-m**), values and error bars represent mean ±SEM, and statistical significance is determined using unpaired two-tailed Student's *t* tests (**c, f, l**) or two-way ANOVA with post-hoc Bonferroni's multiple-comparison analysis (**k, m**). **$P < 0.01$; ***$P < 0.001$; ****$P < 0.0001$. Source data are provided as a Source Data file.

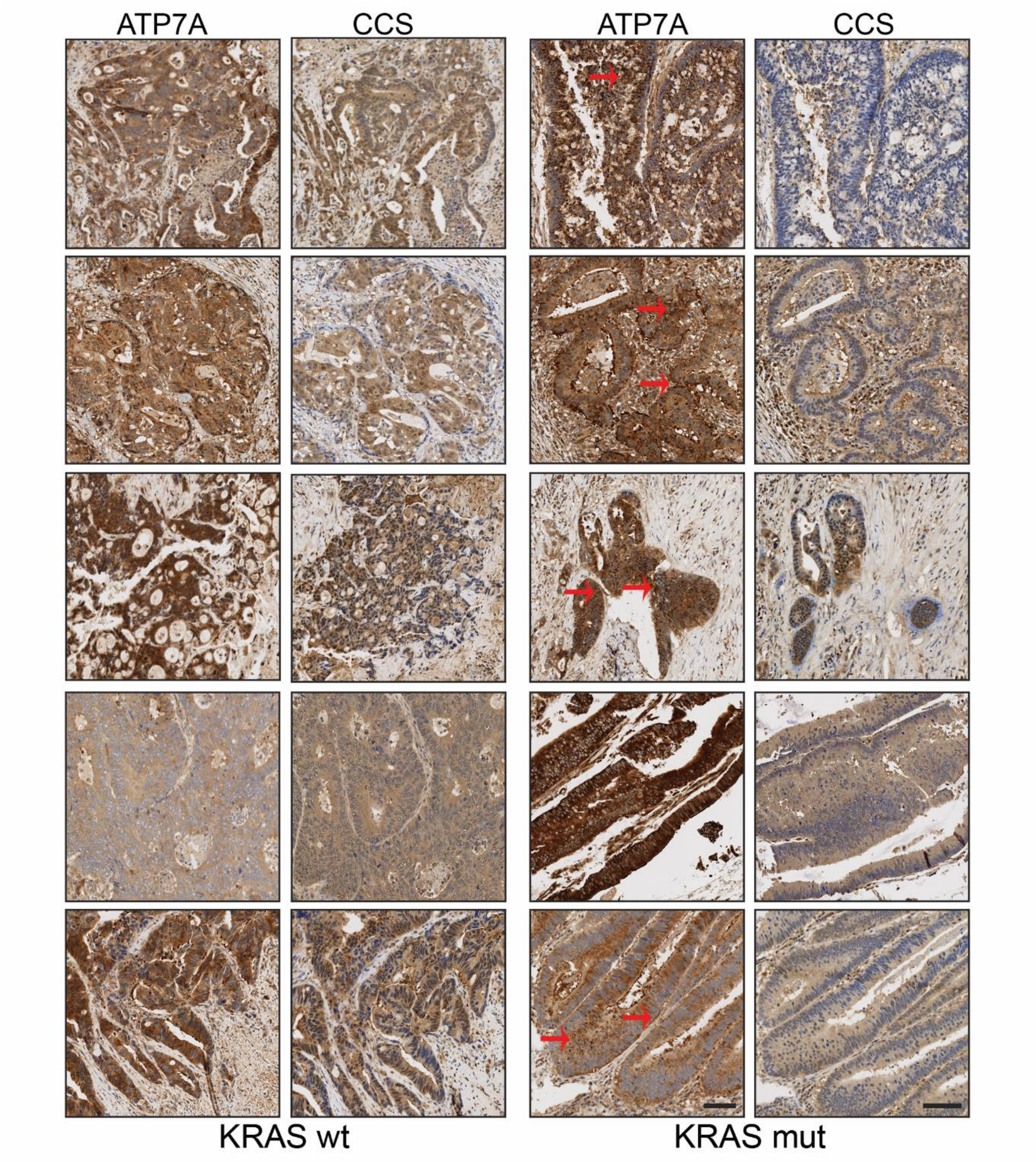

Supplementary Figure 6: ATP7A and CCS levels in KRAS wild-type and KRAS-mutant CRC tumors. Representative IHC images of ATP7A (left) and CCS (right) levels in patient-derived CRC tissues with wild-type KRAS (wt) or mutant KRAS (mut) [n=5 patient tissues per

condition], scale=250 µm. Red arrows indicate granular staining of ATP7A in trans-golgi network or basolateral membrane (or cell-surface) localization of ATP7A.

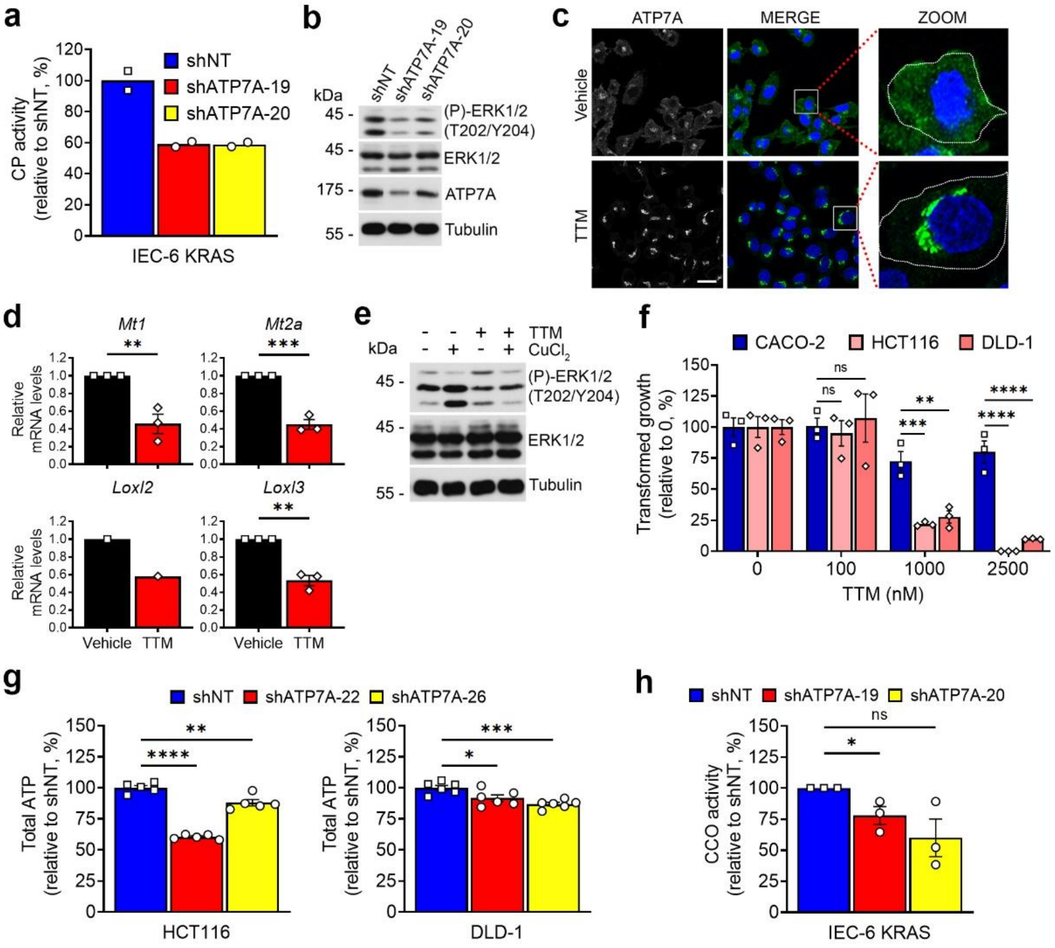

Supplementary Figure 7: ATP7A is required for growth and Cu-dependent functions in KRAS mutant cells. a, CP activity in KRAS cells with NT or ATP7A shRNAs (19 and 20). Data is expressed as mean of n=2 independent experiments. **b,** IB of ATP7A, Tubulin, total and phosphorylated ERK1/2 in lysates of KRAS cells with NT or ATP7A shRNAs (19 and 20). Data represent n=3 independent experiments. **c,** KRAS cells were treated with or without TTM for 24h, and ATP7A (green) was assessed by IF with DAPI (blue). White dashed lines indicate cell boundaries. Data represent n=3 independent experiments, scale=20 µm. **d,** Graph depicting relative mRNA levels of Cu-dependent genes in KRAS cells treated with or without TTM for 24h. Data are representative n=3 independent experiments with two technical replicates. **e,** IB of total

and phosphorylated ERK1/2 and Tubulin in lysates of KRAS cells untreated, treated with TTM or $CuCl_2$, or both for 24h. Data is representative of n=2 independent experiments. **f,** Adherence-independent growth on soft agar of CACO-2, HCT116 and DLD-1 cells treated with the indicated doses of TTM. Data represent n=3 independent replicates per condition, ***P=0.0008 (CACO-2 *versus* HCT116-1000); **P=0.0027 (CACO-2 *versus* DLD-1-1000); ****P<0.0001 (CACO-2 *versus* HCT116-2500 and CACO-2 *versus* DLD-1-2500). **g,** Total ATP levels was measured by luminescence assay in HCT116 (left) and DLD-1 (right) cells with NT or ATP7A shRNAs (22 and 26). Data represent n=5 independent replicates, **P=0.0053 and ****P<0.0001 (left); *P=0.0287 and ***P=0.0003 (right). **h,** As in **a,** except that CCO activity was measured in mitochondrial lysates. Data represent n=3 independent experiments, *P=0.0372. For panels (**d, f-h**), data and error bars represent mean ±SEM, and statistical significance is calculated using unpaired two-tailed Student's t tests (**d, g-h**) or two-way ANOVA with post-hoc Bonferroni's multiple-comparison analysis (**f**). ns: not significant; *P < 0.05; **P < 0.01; ***P < 0.001; ****P < 0.0001. Source data are provided as a Source Data file.

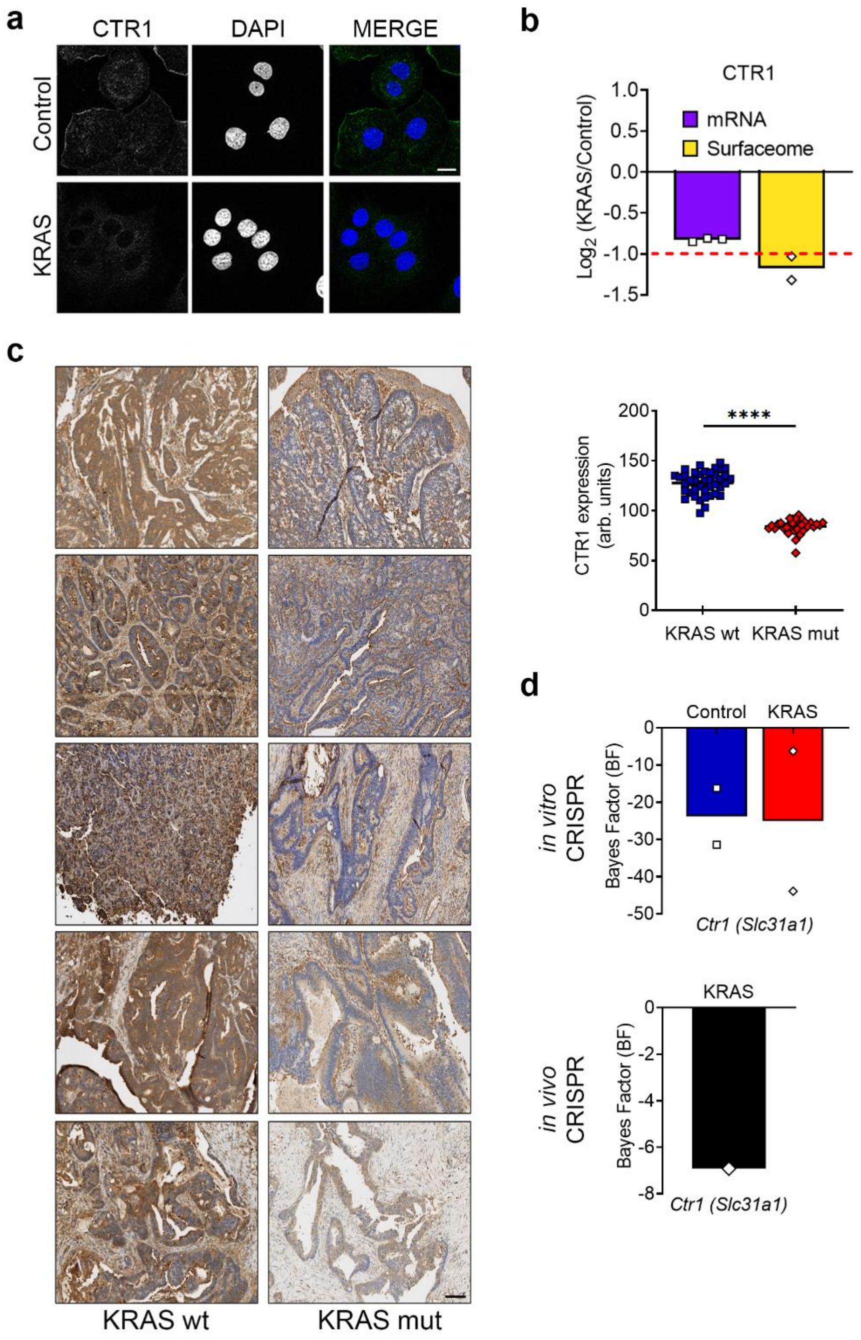

Supplementary Figure 8: CTR1 is downregulated in KRAS-mutant IEC-6 cells and CRC tissues. a, KRAS *versus* Control cells were examined by IF for CTR1 (green) and DAPI (blue), scale=17 µm. **b,** Graph indicating the Log_2 FC (KRAS/Control) from the transcriptome (purple) and surfaceome (yellow) datasets for CTR1. Red dashed line indicates cut-off value for statistical

significance. Data represent n=3 (mRNA) or n=2 (surfaceome) independent replicates per condition. **c,** Representative images (left) for CTR1 staining from IHC profiling of KRAS wt *versus* KRAS mut CRC patient tumors, scale=250 µm. Scatter dot plots for CTR1 levels quantified from IHC images in the indicated conditions (right). Data represent mean ±SEM of n=40 and n=32 random images from five KRAS wt and KRAS mut patient tissues, respectively. Statistical significance is determined using unpaired two-tailed Student's *t* test. ****$P < 0.0001$ **d,** Bar charts representing the BF score of *Ctr1* (*Slc31a1*) from CRISPR/Cas9 screens in KRAS *versus* Control IEC-6 cells (top) and in KRAS IEC-6 xenografts (bottom). Source data are provided as a Source Data file.

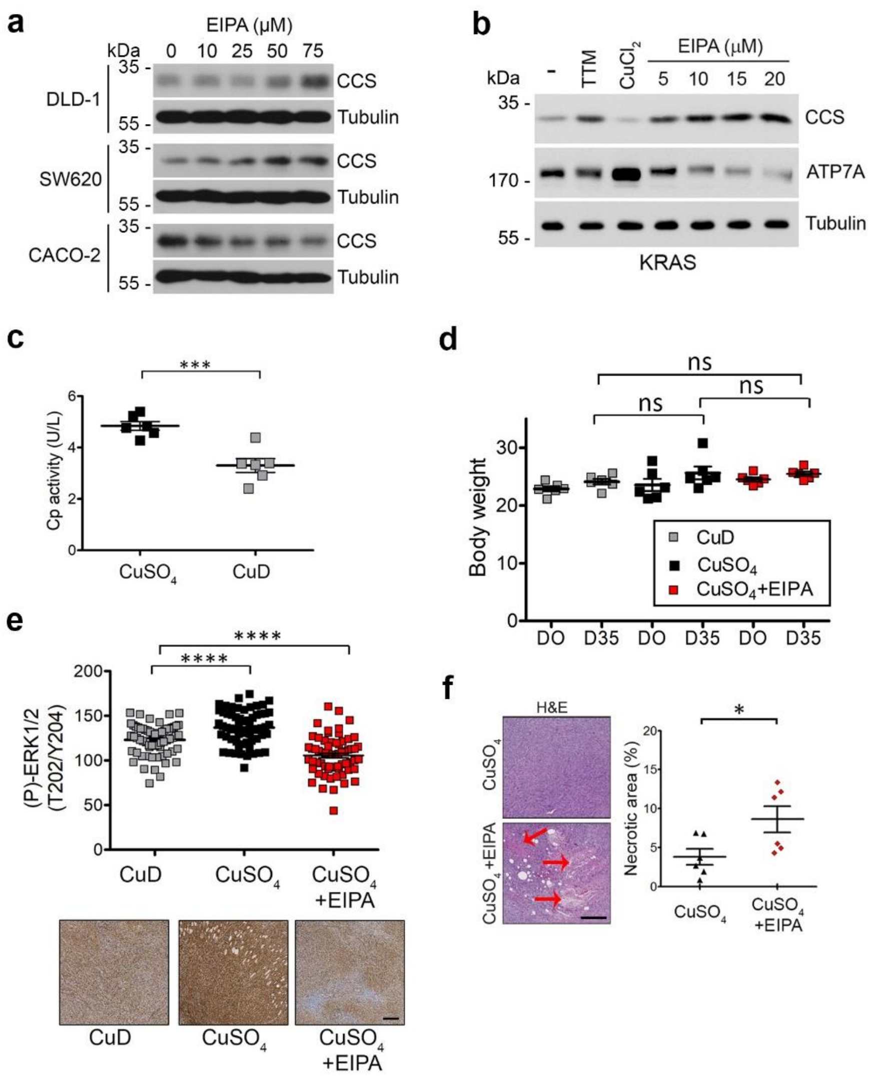

Supplementary Figure 9: Macropinocytosis modulates Cu levels and validation of the *in vivo* tumor growth protocol. a, Whole-cell lysates were collected from CRC cells after treatment with indicated doses of EIPA for 15 min, and CCS and Tubulin levels were analyzed by IB. Data represent n=3 independent experiments. **b,** Whole-cell lysates of KRAS cells were collected after treatment with EIPA at indicated doses, TTM (10 µM) and $CuCl_2$ (100 µM) for 24h. CCS, ATP7A and Tubulin levels were analyzed by IB. Data represent n=3 independent experiments. **c,** Serum was collected from n=6 mice at the end of the study and CP activity was analyzed in two technical replicates for the indicated conditions, ***P=0.0004. **d,** Body weights were recorded in n=6 mice

per condition, for the pre-treatment (D0) and post-treatment (D35) timepoints. **e-f,** Representative IHC images and quantification of phosphorylated-ERK1/2 levels (**e**) and necrotic areas (**f**) for the indicated conditions [n=63 random images from six mice per condition (**e**), ****$P < 0.0001$; n=12 images from six mice per condition (**f**), *P=0.034], scale=250 µm. For panels (**c-f**), values and error bars represent mean ±SEM, and statistical significance is determined using unpaired two-tailed Student's *t* tests. ns: not significant, *$P < 0.05$, ***$P < 0.001$, ****$P < 0.0001$. Source data are provided as a Source Data file.

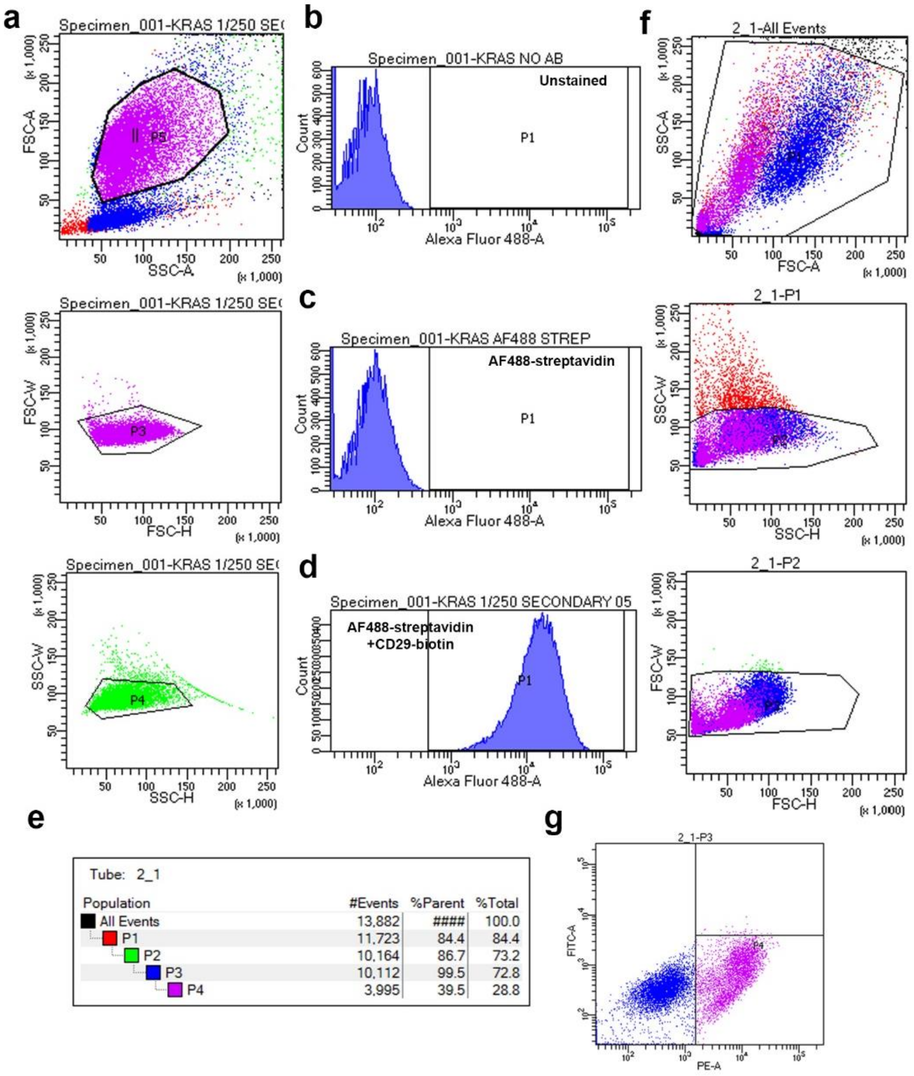

Supplementary Figure 10: Gating strategy for flow cytometry. a, For figure 3, P5 is first selected from FSC-A *versus* SSC-A plot and doublets are removed from P5 population after gating for P3 (in purple) in FSC-W *versus* FSC-H plot, and P4 (in green) from SSC-W *versus* SSC-H plot. **b-d,** From P4, histograms (in blue) is generated and P1 is gated considering negative population in **b)** "Unstained" control without any antibody, and in **c)** "Alexa fluor (AF488)-streptavidin" control with secondary antibody but without primary antibody. **d,** Represents positive population with primary antibody (CD29-biotin) and secondary AF488-streptavidin antibody. **e,** Represents gating strategy used for supplementary figure 5k & 5m. **f,** Population is selected from

FSC-A *versus* SSC-A plot as P1. From P1, doublets are removed by selecting P2 from SSC-W *versus* SSC-H plot and P3 from FSC-W *versus* FSC-H plot. **g,** From P3 a dot plot is generated by plotting FITC-A *versus* PE-A, ANNEXIN-V+ cells are determined by gating for P4.

Supplementary Table 1. Sequences of gene-specific primers for qRT-PCR used in this study.

GENE NAME	SPECIES	PRIMER SEQUENCE (5' – 3')
ATP7A	HUMAN	f-atttaaggcgggaagaagga<br>r-tctgctatcattgggggttg
Atp7a	RAT	f-tgtccccttttattgtgtctacctg<br>r-gcgctttgtaagcctgaatg
Cdh1	RAT	f-gatcctggccctcctgat<br>r-tctttgaccaccgttctcct
Chd2	RAT	f-ccatcatcgcgatacttctg<br>r-ccataccacgaacatgagga
Cd44	RAT	f-gtcatcaaacagaaagcaagga<br>r-aagtcttccaccaaatgggtatt
Egfr	RAT	f-tgcaccatcgacgtctacat<br>r-aactttgggcggctatcag
Mt1	RAT	f-actgcaaatgcacctcctg<br>r-ggcacctttgcagacacag
Mt2a	RAT	f-tgcacctcctgcaagaaaa<br>r-cacttgtccgaagcctcttt
Loxl2	RAT	f-gcttcggcacagctaaagag<br>r-acctcgttgaggtggatgg
Loxl3	RAT	f-gtttctcagactccaacgtcatt<br>r-ccacagctggtcggagtc
Slc31a1	RAT	f-ggaaccatccttatggagacac<br>r-gaggtgggggaagctcag
Tlr4	RAT	f-ggatgatgcctctcttgcat<br>r-tgatccatgcattggtaggtaa
Epha2	RAT	f-agaaggtcattggagcagga<br>r-tgatagccaccggtatctcc
Itgb1	RAT	f-ctgcttctaaagttgcgatcag<br>r-tccataaggtagtacagatcaataggg
Tgfbr1	RAT	f-aaggccaaatattcccaaca<br>r-attttggccatcactctcaag
