# Supplementary material for: Copper bioavailability is a KRAS-specific vulnerability in colorectal cancer
Source: Nat Commun. 2020 Jul 24;11:3701. doi: 10.1038/s41467-020-17549-y (PMC7381612; doi:10.1038/s41467-020-17549-y)
Supplement: Supplementary file 3 — Description of Additional Supplementary Files [file 41467_2020_17549_MOESM3_ESM.pdf]

Description of Additional Supplementary Files

Copper bioavailability is a KRAS-specific vulnerability in colorectal cancer

Aubert *et al.*

File Name: **Supplementary Data 1**
Description: **Transcriptome of IEC-6 ±KRAS^{G12V} analyzed by genome-wide RNA-sequencing. List of up- and downregulated genes expressed as Log_2 (FC KRAS/Control).**

File Name: **Supplementary Data 2**
Description: **Surfaceome of IEC-6 ±KRAS^{G12V} analyzed by Cell Surface Biotinylation (CSB) or Cell Surface Capture (CSC). List of up- and downregulated cell-surface proteins expressed as Log_2 (FC KRAS/Control). Only proteins identified with a false discovery rate (FDR) < 0.5 were considered for analysis, and statistical significance was determined using unpaired one-tailed Student's *t* test, $P < 0.05$. Coverage (%), #total peptides, #unique peptides are also provided. Full data was deposited in ProteomeXchange through partner MassIVE as a complete submission and assigned MSV000085560 and PXD019625. It can be downloaded from ftp://MSV000085560@massive.ucsd.edu.**

File Name: **Supplementary Data 3**
Description: **Correlation between Transcriptome (Supplementary Data 1) and Surfaceome (Supplementary Data 2) datasets. Only proteins whose cell-surface expression is significantly altered by KRAS^{G12V} (unpaired one-tailed Student's *t* test, $P < 0.05$) were considered for the correlation.**

File Name: **Supplementary Data 4**
Description: **List of all gRNAs accounting for the KRAS-library used in the *in vitro* and *in vivo* CRISPR/Cas9 screens.**

File Name: **Supplementary Data 5**
Description: **List of genes targeted with KRAS-library gRNAs in the two *in vitro* CRISPR/Cas9 screens, with associated log Bayes Factor (BF) for Control and KRAS IEC-6 cells, and differential essentiality scores (Z-score).**

File Name: **Supplementary Data 6**
Description: **List of genes targeted with KRAS-library gRNAs in the *in vivo* CRISPR/Cas9 screens, with associated log Bayes Factor (BF).**
